# Serratus posterior superior intercostal plane block versus thoracic paravertebral block for pain management after video-assisted thoracoscopic surgery: a randomized prospective study

**DOI:** 10.1016/j.bjane.2025.844647

**Published:** 2025-06-03

**Authors:** Güvenç Doğan, Onur Küçük, Selçuk Kayır, Gökçe Çiçek Dal, Bahadır Çiftçi, Musa Zengin, Ali Alagöz

**Affiliations:** a Hitit University Faculty of Medicine, Department of Anesthesiology and Reanimation, Çorum, Turkey; b University of Health Sciences, Ankara Ataturk Chest Diseases and Thoracic Surgery Training and Research Hospital, Department of Anesthesiology and Reanimation, Ankara, Turkey; c Siirt Training and Research Hospital, Department of Anesthesiology and Reanimation, Siirt, Turkey; d Istanbul Medipol University, Department of Anesthesiology and Reanimation, Istanbul, Turkey; e Ankara Etlik City Hospital, Department of Anesthesiology and Reanimation, Ankara, Turkey

**Keywords:** Nerve block, Pain, Serratus posterior superior intercostal plane block, Thoracic paravertebral block, Video-assisted thoracic surgery

## Abstract

**Background:**

Video-Assisted Thoracoscopic Surgery (VATS) is a minimally invasive procedure associated with faster recovery and fewer complications compared to open thoracotomy. Effective postoperative pain management is important for optimizing recovery. This study compares the analgesic efficacy of the Serratus Posterior Superior Intercostal Plane Block (SPSIPB) and Thoracic Paravertebral Block (TPVB) for postoperative pain following VATS.

**Methods:**

In this randomized, prospective, double-blind study, 70 patients aged 18–65 years (ASA I–III) undergoing VATS were randomly assigned to Group TPVB (n = 35) or Group SPSIPB (n = 35). The primary outcome was the 24-hour postoperative Visual Analog Scale (VAS) pain score at rest. Secondary outcomes included VAS pain scores during coughing, time to first opioid request, total opioid consumption within 24 hours, patient satisfaction, and Quality of Recovery-15 (QoR-15) scores. Opioid consumption was assessed using intravenous tramadol through Patient-Controlled Analgesia (PCA), with additional morphine, if required.

**Results:**

The mean age of the patients was 52 ± 11 years, and 64.2% were male. VAS pain scores were evaluated at 24 hours and at seven time points. There was no significant difference between groups (p > 0.05) except at 1 hour postoperatively, where the TPVB group had a significantly lower resting VAS score (19 [8–28] vs. 26 [18.5–33], p = 0.031). The total 24 hour tramadol consumption was 220 mg (135–260) in the TPVB group versus 150 mg (110–230) in the SPSIPB group (p = 0.129). The proportion of patients requiring additional analgesia was 25.7% in the TPVB group versus 28.5% in the SPSIPB group (p = 0.788). Preoperative and postoperative QoR-15 scores were similar between the groups (preoperative: 137 vs. 136, p = 0.878; postoperative: 133 vs. 132, p = 0.814). Patient satisfaction scores were also comparable (8 [7–10] vs. 9 [7–10], p = 0.789).

**Conclusion:**

SPSIPB provides analgesic efficacy similar to TPVB for VATS, with comparable pain scores, opioid consumption, and recovery outcomes. Given its ease of use and safety profile, SPSIPB represents a promising alternative to TPVB in multimodal analgesia for minimally invasive thoracic surgery.

## Introduction

Surgical intervention via Video-Assisted Thoracoscopic Surgery (VATS) is accomplished through the utilization of two to three incisions (2–3 cm) in the skin, accompanied by the deployment of an endo-camera and surgical instruments within the thoracic cavity. In recent years, VATS has gained prominence as the conventional minimally invasive surgical technique for pulmonary procedures.^1^ Compared to open thoracotomy, VATS offers advantages such as expedited recovery, reduced hospital stays, and a lower risk of complications.^2^ Although VATS is considered less painful than thoracotomy, both acute and chronic pain remain significant concerns following VATS surgery.^3^ Thoracic Epidural Analgesia (TEA), the gold standard for pain management after thoracotomy,^4^ is less commonly used for analgesia following VATS. However, given the relatively limited nature of pain associated with VATS, thoracic wall blocks may be more effective for this patient population.^3^^,^^5^ The challenges associated with TEA, including difficulties in application and side effects such as hypotension, urinary retention, and nausea/vomiting, have led to the increasing acceptance of less invasive analgesic techniques for minimally invasive surgery.^6^

In recent years, regional block techniques have become an indispensable component of multimodal analgesia for postoperative pain management. Thoracic Paravertebral Block (TPVB), Erector Spinae Plane Block (ESPB), and Serratus Anterior Plane Block (SAPB) are commonly used as regional anesthesia procedures in thoracic surgery.^1^^,^^5^ TPVB has long been established in the literature as the first-line regional technique for VATS surgery.^7^ In addition, the Serratus Posterior Superior Intercostal Plane Block (SPSIPB), performed under Ultrasound (US) guidance, was described by Tulgar et al.^8^ in 2023 and has since become a routine interfascial plane block for suitable patients undergoing thoracic surgery.^9^ This block involves injection between the serratus posterior superior muscle and the rib at the level of the second or third rib.^10^ The SPSIPB has been shown to provide analgesia for a range of conditions, including interscapular pain, chronic myofascial pain syndromes, scapulocostal syndrome, and shoulder discomfort.^10^ The serratus posterior superior muscle attaches to the lateral edges of the second to fifth ribs and is located between the C7 and T2 vertebral levels. It receives its innervation primarily from the ventral rami of the upper intercostal nerves (T2–T5) and the lower cervical spinal nerves, reflecting its anatomical location between the cervical and upper thoracic vertebral levels.^10^ The potential of SPSIPB to effectively target these nerves has been demonstrated by Tulgar et al. in a cadaver study, which demonstrated the efficacy of SPSIPB in providing analgesia for thoracic procedures, including persistent myofascial pain, breast surgery, thoracic surgery, and shoulder surgery.^8^

To date, no randomized trials have been reported in the literature comparing the efficacy of SPSIPB with TPVB for postoperative analgesia following VATS. This study aimed to evaluate the postoperative analgesic efficacy of ultrasound guided SPSIPB compared to TPVB in patients undergoing VATS, based on the hypothesis that SPSIPB is non-inferior to TPVB.

## Materials and methods

### Study design and patients

This study is a two-center, prospective, randomized, double-blind, and observational trial. After obtaining approval from the Ethics Committee of the Faculty of Medicine at Hitit University (approval n° 2023-181), 70 patients scheduled for VATS surgery were included in the study. Inclusion criteria were patients aged 18–65 years, with an American Society of Anesthesiologists (ASA) physical status classification of I–III, a Body Mass Index (BMI) of < 35 kg.m^-2^, and who had read and signed the informed consent form. The study was registered on ClinicalTrials.gov with reference number NCT06219369 (January 23, 2024). The recruitment period was between January 31, 2024, and August 15, 2024, and included patients who underwent surgery at both Ankara Ataturk Sanatorium Training and Research Hospital and Hitit University Erol Olcok Training and Research Hospital.

Patients were excluded from the study if they could not communicate in Turkish, declined consent, had technical problems with the Patient-Controlled Analgesia (PCA) device, or were unable to use the Visual Analog Scale (VAS) or complete the Quality of Recovery-15 (QoR-15) questionnaire. Other exclusion criteria included allergy to local anesthetics or study-specific analgesics; pregnancy or breastfeeding; uncontrolled anxiety or substance dependence; history of thoracic surgery, trauma, neuromuscular or peripheral nerve disorders; diabetes mellitus, hepatic or renal insufficiency, or coagulation abnormalities; chronic opioid or steroid use; widespread pain; anticoagulant therapy; infection at the block insertion site; early termination of surgery; or no planned postoperative extubation.

**Figure 1:**
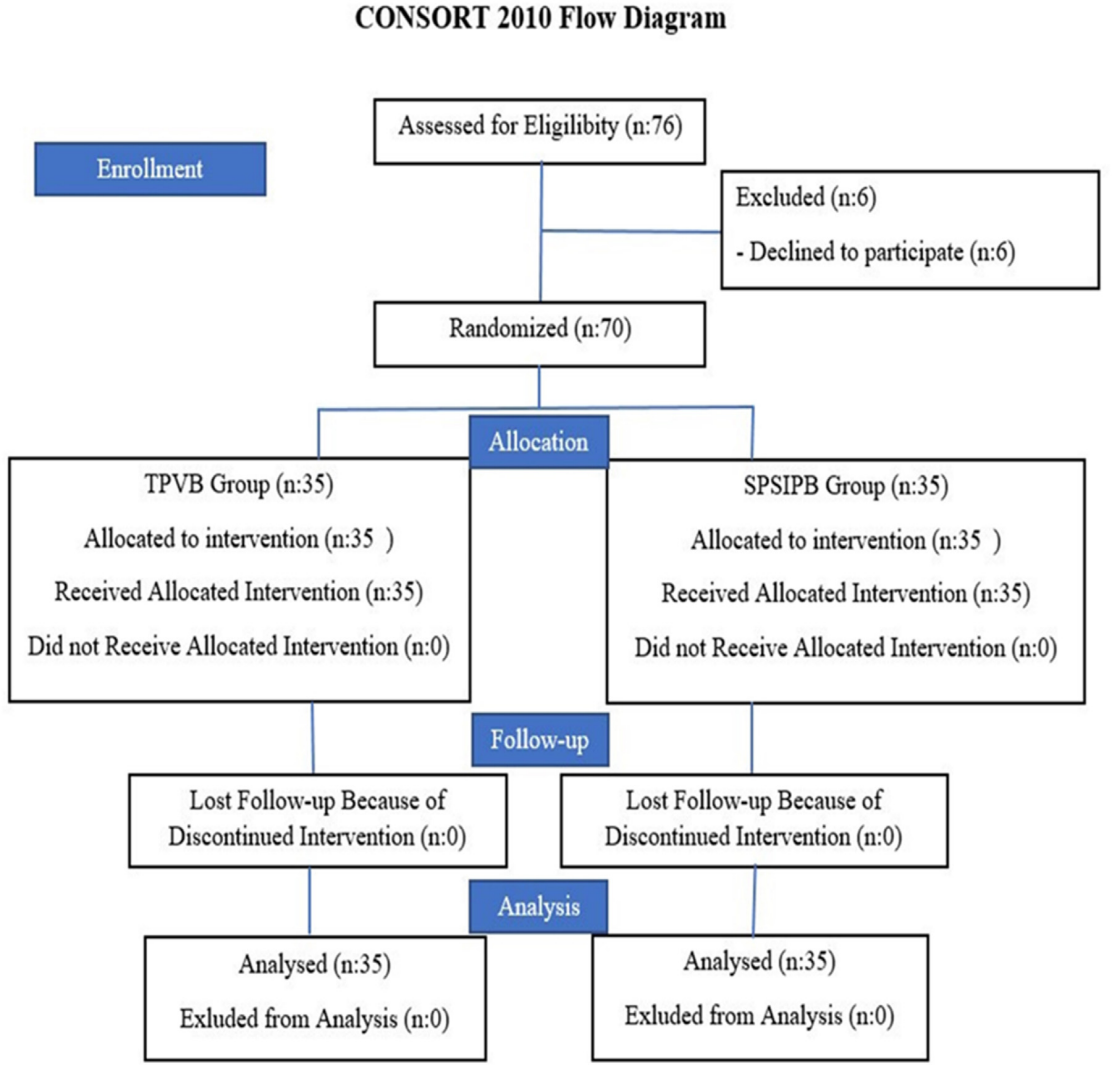
Consort flow chart. TPVB, Thoracic Paravertebral Block; SPSIPB, Serratus Posterior Superior Intercostal Plane Block.

Patient enrollment and allocation followed the Consolidated Standards of Reporting Trials (CONSORT) flow chart, as illustrated in Figure 1. Patient confidentiality was protected in accordance with the Declaration of Helsinki.

### Interventions

Patients enrolled in the study were randomly assigned to the TPVB and SPSIPB groups using a computer-generated randomization table prepared by a researcher not involved in the study. To ensure blinding, each patient was assigned a random code, which was placed in a sealed envelope. The anesthetist in the operating room retrieved the appropriate sealed envelope from a file, specifying the block to be administered to each randomized patient. The patient, surgeon, and individuals overseeing postoperative pain management were unaware of the patient’s group assignment. During the preoperative examination, the patients were educated on pain assessment and the implementation of Patient-Controlled Analgesia (PCA). Standard anesthesia monitoring, including noninvasive arterial blood pressure monitoring, heart rate monitoring, electrocardiography, and peripheral oxygen saturation testing, was performed once the patients were admitted to the operating room. A 20G catheter was inserted to establish intravenous access, and the time of anesthesia initiation was recorded. Premedication was 0.03 mg.kg^-1^ of midazolam, followed by induction of anesthesia with 2 mg.kg^-1^ of propofol and 1 mcg.kg^-1^ of fentanyl after preoxygenation. After administering 0.6 mg.kg^-1^ of rocuronium bromide Intravenous (IV) for muscle relaxation, intubation was performed using a left double-lumen endotracheal tube. All patients underwent radial artery cannulation for arterial monitoring and lung-protective single-lung ventilation. Mechanical ventilation was performed with a target end-tidal CO_2_ of 35–40 mmHg. A mixture of O_2_/air (FiO_2_ = 0.50), sevoflurane (minimum alveolar concentration 0.8–1), and an IV infusion of remifentanil (adjusted according to the patient’s hemodynamic data) was used to maintain anesthesia. The remifentanil infusion was planned in a dose range of 0.01‒0.2 mcg.kg^-1^.min^-1^ to maintain the patient's mean arterial blood pressure within 20% of baseline. Thirty minutes before the end of surgery, all patients received 1g of paracetamol and 1 mg.kg^-1^ of tramadol for analgesia, along with 4 mg of ondansetron IV for nausea and vomiting prophylaxis. Following the conclusion of the surgical procedure and the closure of the skin incision, regional anesthesia was administered. After the specified block procedure was completed, general anesthesia was terminated, and the neuromuscular blockade was reversed with 4 mg.kg^-1^ of sugammadex. Once adequate respiratory effort was observed, patients were extubated. Postoperatively, patients were transferred to the intensive care unit for close monitoring and advanced surveillance.

The duration of the regional anesthesia, the time the block was performed, the end time of surgery, and the end time of anesthesia were all noted. The blocks were performed by anesthesiologists experienced in ultrasound and the routine application of blocks in clinical practice.

### Block procedures applied

#### Thoracic paravertebral block

**Figure 2:**
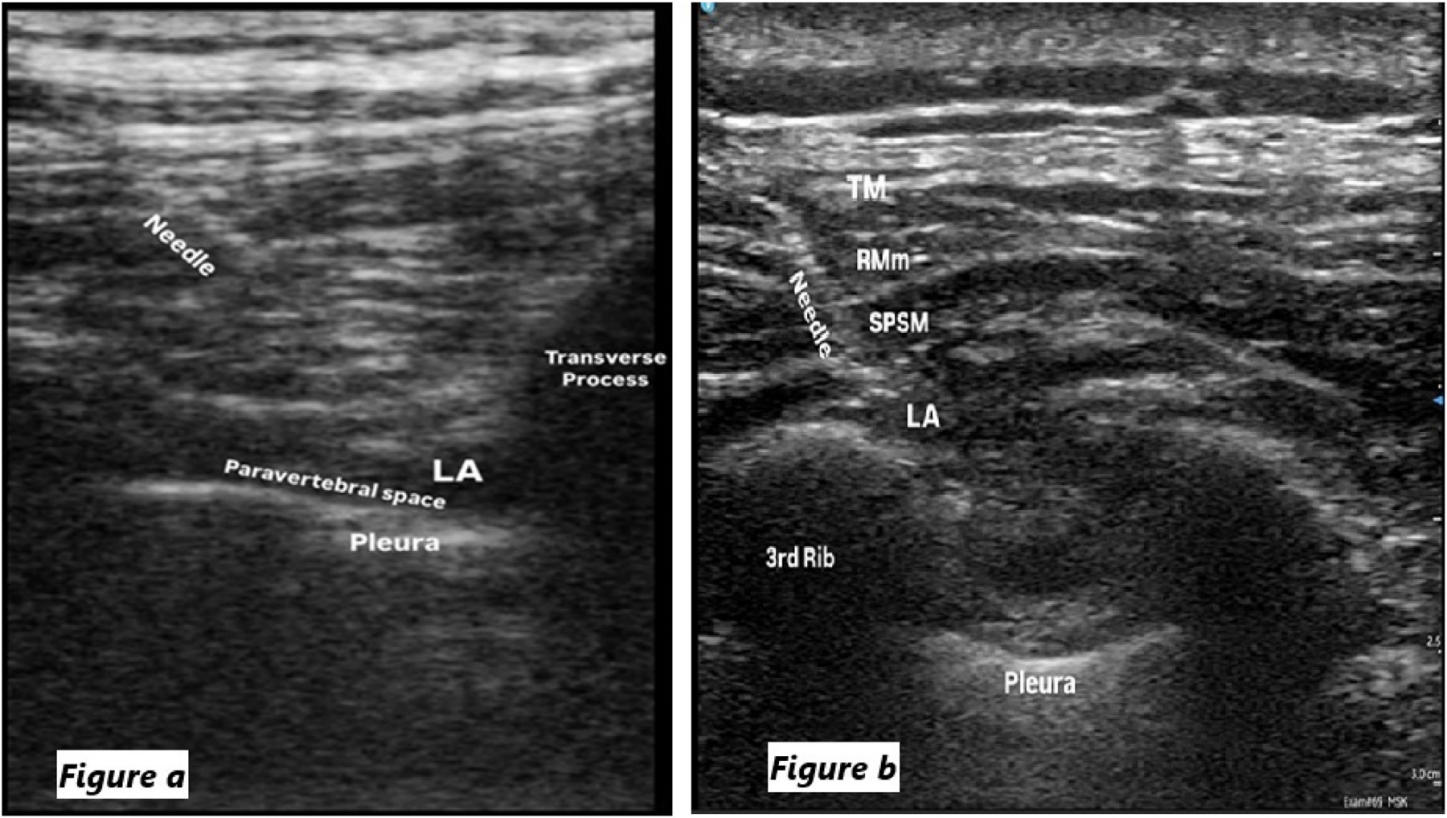
(a) Sono-anatomy and spread of LA during Thoracic paravertebral block (LA, Local Anesthetic). (b) Sono-anatomy and spread of LA during SPSIPB (Tm, Trapezius muscle; RMm, Rhomboid Major muscle; SPSN, Serratus Posterior Superior muscle; LA, Local Anesthetic).

The procedure was performed using an 80 mm peripheral block needle (Braun 360°) with the patient in the lateral decubitus position in accordance with the guideline definitions.^11^ A high-frequency sterile ultrasound linear probe (6–13 MHz) was placed 2–3 cm lateral to the T5 spinous process. After visualizing the transverse process, the muscular structures up to the transverse process, the paravertebral space, the internal intercostal membrane, and the pleura, the needle was advanced using the in-plane technique until it reached the paravertebral space. After confirming the accuracy of needle placement with the transverse technique, 30 mL of 0.25% bupivacaine was injected into this area (Fig. 2a).

#### Serratus posterior superior intercostal plane block

As described by Tulgar et al.,^8^ the block was performed after completion of the surgical procedure but before the patient was awakened. A high-frequency sterile linear ultrasound probe (6–13 MHz) and an 80 mm block needle (Braun 360°) were used. The procedure was performed with the patient in the lateral decubitus position. After slight lateral displacement of the scapula, the spine of the scapula was visualized with ultrasound and the probe was moved medially. Once the end of the scapular spine was located, the probe was placed sagittally at the superior angle of the scapula and the third rib was visualized. The block needle was advanced in the craniocaudal direction, entering between the serratus posterior superior muscle and the third rib. A 2 mL saline injection was administered to confirm the correct placement of the block. After confirming the block site, 30 mL of 0.25% bupivacaine was injected (Fig. 2b).

### Postoperative analgesia protocol and pain assessment

Postoperative pain monitoring was performed by a blind pain assessment nurse or the anesthetist responsible for postoperative pain management, who was unaware of the patient’s group allocation. VAS was used to assess the patient’s perception of pain, which was converted into a numerical format (scaled from 0 to 100 mm; 0 = no pain and 100 mm = unbearable pain). The VAS score was evaluated under both resting and active movement conditions (e.g., during coughing). VAS scores were recorded at 0, 1, 3, 6, 12, 18, and 24 hours postoperatively.

All patients received IV paracetamol at a dosage of 1 g every 8 hours, with postoperative analgesia provided through PCA using IV tramadol. Our PCA protocol was designed to deliver a 10 mg bolus of tramadol on demand without baseline infusion, with a maximum dose of 400 mg/day and a lockout period of 20 minutes. Tramadol consumption was recorded for intervals of 0–1 hour, 1–12 hours, 12–24 hours, and a total of 24 hours. During the pain monitoring periods, intravenous morphine was administered as a slow infusion at a dose of 0.05 mg.kg^-1^ to patients with a VAS score of 40/100 mm or above and the number of applications was documented. In addition, the total morphine consumption was converted to tramadol equivalents (morphine consumption in mg × 10 = tramadol in mg)^12^ and added to the total tramadol consumed during the patient’s follow-up period by PCA.

The time of the patient’s first postoperative opioid analgesic requirement and the total amount of opioid analgesics administered during the first 24 hours were recorded. Nausea and vomiting during the first 24 hours were monitored using the Postoperative Nausea and Vomiting (PONV) scale: PONV1 (no nausea or vomiting), PONV2 (nausea present, no vomiting), PONV3 (one episode of vomiting or persistent nausea), PONV4 (two or more vomiting episodes or severe/continuous retching). Patients with a nausea score of 2 or higher received 4 mg of ondansetron via IV infusion. The total dose of ondansetron administered over 24 hours was recorded.

To evaluate the quality of postoperative recovery, patients completed the QoR-15 scale, a self-reported questionnaire, twice: once in the waiting area on the morning of surgery and again 24 hours postoperatively. Patient demographics were recorded before surgery, while postoperative data included the time of first oral intake, time to gas/stool passage, and the duration until the first mobilization (unassisted standing).

### Outcomes

The primary outcome measure was the postoperative 24-hour VAS pain score in the TPVB and SPSIPB groups. Other outcome measures included resting and cough VAS pain scores between the two groups, opioid consumption during the first 24 hours postoperatively, side effects associated with opioid use (such as allergic reaction, nausea, and vomiting), patient satisfaction at 24 hours postoperatively, and preoperative and postoperative QoR-15 scale scores.

### Sample size

The sample size for this study was calculated using G*Power software, version 3.1.9.6. The effect size employed in the calculation was derived from the study by Qiu et al., which compared SAPB with a single injection of 30 mL local anesthetic to TPVB.^13^ The study by Qiu et al.^13^ reported that the mean 24-hour resting VAS score for TPVB was 19 ± 11 mm and the mean cough VAS score was 35 ± 14 mm. The minimum clinically significant change in pain as measured by VAS was determined to be 13 mm, which is widely accepted in the literature.^14^ Consequently, to detect a minimum difference of 13 mm between the SPSIPB and TPVB groups, a minimum sample size of 27 was calculated for each treatment arm, with a type-1 error level of 0.05 and a study power of 90% (effect size: 0.9). Given that SPSIPB is a novel block, a margin of error of approximately 20% was added for each treatment arm to account for potential deviations from the protocol. It was determined that 35 patients would be included in each treatment arm.

### Statistical analysis

Statistical analyses were performed using SPSS for Windows, version 22.0 (SPSS Inc., Chicago, IL, USA). The Shapiro-Wilk test was used to evaluate the normality of the distribution of continuous variables, while the Levene test was applied to assess the homogeneity of variances. Continuous variables were presented as mean ± Standard Deviation (SD) for normally distributed data and as median (Q1–Q3) for non-normally distributed data, unless otherwise specified. Categorical variables were expressed as numbers and percentages [n (%)]. Comparisons between two independent groups were made using Student’s *t*-test for normally distributed continuous variables and the Mann-Whitney *U* test for non-normally distributed variables. Categorical variables were compared using Pearson’s Chi-Square test; however, when the expected frequency in any cell of the contingency table was less than 5, Fisher’s exact test was used to ensure the validity of the results. Missing data were handled by complete case analysis, and no imputation methods were used. A p-value of less than 0.05 was considered statistically significant.

Graphical representations were generated using Jamovi version 2.3.21.0 software (Sydney, Australia).

## Results

Between January 31, 2024, and August 15, 2024, a total of 76 patients were screened for eligibility at two participating centers. After the exclusion of six patients who declined to participate, 70 patients were randomized and included in the final analysis, with 35 patients in the TPVB group and 35 patients in the SPSIPB group (Fig. 1).

The mean age of the patients was 52 ± 11 years, and 64.2% (n = 45) were male. Table 1 presents the distribution of age, gender, BMI, ASA classification, comorbidities, anesthesia duration, and surgical duration by group. There were no statistically significant differences between the two groups regarding these parameters. Table 2 displays the VAS scores during rest and coughing at different time points. When comparing the VAS rest scores between the groups, no statistically significant differences were observed (0 hours, p = 0.688; 3 hours, p = 0.282; 6 hours, p = 0.571; 12 hours, p = 0.564; 18 hours, p = 0.934; 24 hours, p = 0.572). However, the VAS rest score at 1 hour was statistically lower in the TPVB group (p = 0.031). In analyzing the VAS cough scores, no statistically significant differences were found between the groups (0 hours, p = 0.948; 1 hour, p = 0.267; 3 hours, p = 0.434; 6 hours, p = 0.902; 12 hours, p = 0.809; 18 hours, p = 0.972; 24 hours, p = 0.737). The median amount of tramadol requested via PCA postoperatively was 200 mg in the TPVB group and 150 mg in the SPSIPB group, with no statistically significant difference in tramadol demand between the groups (p = 0.183, Table 3). Regarding requests for additional analgesia, 9 (25.7%) patients in the TPVB group and 10 (28.5%) patients in the SPSIPB group requested it within the first 24 hours postoperatively (p = 0.788). There was no statistically significant difference in the amount of additional analgesia consumed between the groups (p = 0.890). The total amount of tramadol consumed within 24 hours was a median of 220 mg in the TPVB group and 150 mg in the SPSIPB group, with no statistically significant difference (p = 0.129, Table 3). When evaluating the total amount of tramadol consumed in the first 6 hours, the TPVB group consumed less, but the difference was not statistically significant (p = 0.307).

**Table 1:** Comparison of demographic data between groups.

	TPVB (n = 35)	SPSIPB (n = 35)	p-value
**Age, year, median (Q_1_ ‒ Q_3_)**	57 (47.0 ‒ 59.5)	58 (44.5 ‒ 61.5)	0.902^b^
**Sex, n (%)**			0.454^δ^
Female	11 (31.43%)	14 (40.0%)	
Male	24 (68.57%)	21 (60.0%)	
**BMI, kg.m^-2^, mean ± SD**	25.81 ± 3.53	26.31 ± 5.02	0.630^a^
**ASA, n (%)**			0.730^δ^
ASA I	4 (11.43%)	2 (5.71%)	
ASA II	17 (48.57%)	17 (48.57%)	
ASA III	14 (40.0%)	16 (45.72%)	
**Comorbidities, n (%)**			0.584^δ^
No	10 (28.57%)	8 (22.86%)	
Yes	25 (71.43%)	27 (77.14%)	
**Anesthesia Procedure Duration (min), mean ± SD**	189.9 ± 55.5	186.3 ± 55.3	0.786^a^
**Surgical Procedure Duration (min), mean ± SD**	159.3 ± 54.3	161.0 ± 54.6	0.897^a^

Categorical variables are expressed as either ^δ^ Frequency (n) or percentage (%), while continuous variables are expressed as ^a^ The mean ± Standard Deviation (SD) or ^b^ The median (Q1; 25 Percentile ‒ Q3; 75 Percentile). Pearson's Chi-Square test or Fisher's exact test were used to compare categorical variables, while the student *t*-test or the Mann-Whitney *U* test were used to compare continuous variables. p-values that are statistically significant are in bold. TPVB, Thoracic Paravertebral Block; SPSIPB, Serratus Posterior Superior Intercostal Plane Block; BMI, Body mass index; ASA, American Society of Anesthesiologists; SD, Standard Deviation; min, minute.

**Table 2:** Comparison of VAS data between groups.

	TPVB (n = 35)	SPSIPB (n = 35)	p-value
	Median (Q1 ‒ Q3)	Median (Q1 ‒ Q3)	
**VAS rest, mm**			
0 hour	17 (2 ‒ 32.5)	20 (8 ‒ 31)	0.688^a^
1 hours	19 (8 ‒ 28)	26 (18.5 ‒ 33)	**0.031**^a^
3 hours	18 (7.5 ‒ 23.5)	20 (14 ‒ 25.50)	0.282^a^
6 hours	16 (7 ‒ 22)	18 (10 ‒ 23)	0.571^a^
12 hours	21 (13 ‒ 25)	22 (15 ‒ 26)	0.564^a^
18 hours	21 (16 ‒ 26.5)	22 (17 ‒ 25)	0.934^a^
24 hours	19 (12 ‒ 23)	16 (10.5 ‒ 24)	0.572^a^
**VAS cough, mm**			
0 hour	31 (15.5 ‒ 46)	32 (16 ‒ 42)	0.948^a^
1 hours	31 (17 ‒ 39.5)	32 (26 ‒ 44)	0.267^a^
3 hours	28 (14.5 ‒ 34)	26 (21.5 ‒ 38)	0.434^βa^
6 hours	26 (16.5 ‒ 32)	25 (16 ‒ 34.5)	0.902^a^
12 hours	28 (22.5 ‒ 32.5)	28 (21 ‒ 34.5)	0.809^a^
18 hours	27 (24 ‒ 33)	30 (24 ‒ 32.5)	0.972^a^
24 hours	26 (19.5 ‒ 32)	24 (18 ‒ 34)	0.737^a^

The ^a^median is used to express continuous variables (Q1; 25 Percentile-Q3; 75 Percentile). The Mann-Whitney *U* test was used for comparisons between continuous variables. p-values that are statistically significant are in bold. TPVB, Thoracic Paravertebral Block; SPSIPB, Serratus Posterior Superior Intercostal Plane Block; VAS, Visual Analog Scale.

**Table 3:** Comparison of analgesic consumption, patient satisfaction, and QoR scores between groups.

	TPVB (n = 35)	SPSIPB (n = 35)	p-value
**PCA Tramadol Consumption, mg, median (Q_1_ ‒ Q_3_)**			
0 ‒ 1 hour	20 (10 ‒ 30)	20 (10 ‒ 30)	0.695^b^
1 ‒ 12 hours	60 (30 ‒ 80)	40 (20 ‒ 65)	0.262^b^
12 ‒ 24 hours	120 (95 ‒ 155)	100 (60 ‒ 125)	0.065^b^
Total	200 (135 ‒ 260)	150 (110 ‒ 230)	0.183^b^
**Request for Additional Analgesia, n (%)**			0.788^a^
No	26 (%74.29)	25 (%71.43)	
Yes	9 (%25.71)	10 (%28.57)	
**Additional Morphine, mg, median (Q_1_ ‒ Q_3_)**	0 (0 ‒ 1.5)	0 (0 ‒ 1.5)	0.890^b^
**Total Tramadol Consumption, mg, median (Q_1_** ‒ **Q_3_)**			
0 ‒ 6 hours	50 (20 ‒ 60)	50 (30 ‒ 80)	0.307^b^
0 ‒ 24 hours	220 (135 ‒ 260)	150 (110 ‒ 230)	0.129^b^
**PONV score, max, n (%)**			0.743^a^
1	34 (97.14%)	32 (91.43%)	
2	0	2 (5.71%)	
3	1 (2.86%)	1 (2.86%)	
**Patient Satisfaction, median (Q_1_ ‒ Q_3_)**	8 (7 ‒ 10)	9 (7 ‒ 10)	0.789^b^
**Preoperative QoR-15 score**	137 (130 ‒ 141)	136 (132 ‒ 142)	0.878^b^
**Postoperative QoR-15 score**	133 (126 ‒ 137.5)	132 (129 ‒ 138)	0.814^b^

Categorical variables are expressed as either ^a^ frequency (n) or percentage (%), while continuous variables are expressed as * the mean ± Standard Deviation (SD) or ^b^ the median (Q1; 25 Percentile ‒ Q3; 75 Percentile). Pearson's Chi-Square test or Fisher's exact test were used to compare categorical variables, while the student *t*-test or the Mann-Whitney *U* test were used to compare continuous variables. p-values that are statistically significant are in bold. TPVB, Thoracic Paravertebral Block; SPSIPB, Serratus Posterior Superior Intercostal Plane Block; QoR-15, Quality of Recovery-15.

**Figure 3:**
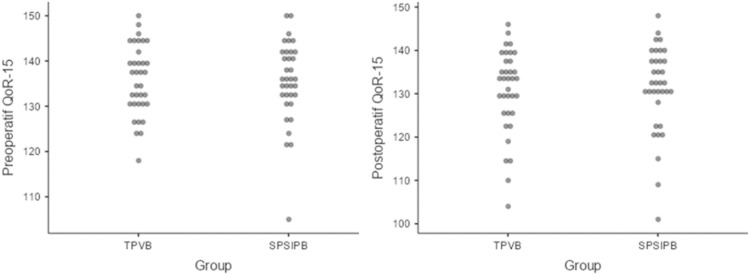
Graphs of preoperative and postoperative QoR-15 scores in the groups (TPVB, Thoracic Paravertebral Block; SPSIPB, Serratus Posterior Superior Intercostal Plane Block; QoR-15, Quality of Recovery-15).

When examining the maximum PONV scores at follow-up for the groups, no statistically significant difference was found (p = 0.743). In the TPVB group, ondansetron was administered to 1 patient (2.8%) within 24 hours, while in the SPSIPB group, it was administered to 3 patients (8.5%) (p = 0.614). After 24 hours of follow-up, patient satisfaction was assessed, and both groups reported high satisfaction levels; the TPVB group had a median score of 8 (7–10), while the SPSIPB group had a median score of 9 (7–10) (p = 0.789). The preoperative and postoperative QoR-15 score changes for the patients are detailed in Figure 3. In the TPVB group, the preoperative QoR-15 score was a median of 137, while at the 24 hour postoperative mark, it was a median of 133. In the SPSIPB group, the preoperative QoR-15 score was a median of 136 and at the end of the 24 hour postoperative period, it was a median of 132. There was no statistically significant difference in the changes in QoR-15 scores between the groups (p-values: preoperative 0.878, postoperative 0.814) (Table 3).

## Discussion

The results of our study evaluating two different thoracic body blocks in patients undergoing VATS indicate that both TPVB and the relatively new plane block, SPSIPB, demonstrate similar analgesic and clinical outcomes.

Minimally invasive thoracic surgery significantly improves patient comfort while limiting potential complications, thus facilitating early discharge. However, despite all these advances in minimally invasive surgery, early postoperative pain and the risk of developing chronic pain due to inadequate management remain current issues.^15^ To address this, multimodal analgesic techniques have significant potential due to their ability to reduce the incidence of side effects and their different mechanisms of action. The contribution of thoracic body blocks, in addition to systemic analgesia, cannot be overlooked. It is well established that TPVB provides effective postoperative analgesia.^16^ There are even studies suggesting that TPVB achieves similar analgesic efficacy in thoracotomies.^17^ Although TPVB is easier to perform compared to TEA, the proximity of the paravertebral space to the pleura and other vascular and nerve structures can complicate the procedure and increase complication rates. Recently, thoracic plane blocks have been widely used in clinical practice due to their ease of application and similar analgesic efficacy.^17^

In 2007, Henrik Kehlet and the Procedure-Specific Postoperative Pain Management (PROSPECT) study group classified both TEA and TPVB as Class A evidence based on randomized clinical trials.^18^ A protocol for managing postoperative pain after thoracotomy, which includes recommendations for regional analgesic methods, is available through PROSPECT. Among the recommended regional analgesic techniques for VATS surgery, TPVB and ESPB ranked first, followed by SAPB.^19^ ESPB has inconsistent distribution in radiological and cadaver studies, which means that different distribution and dermatomal involvement may occur in each patient. In this regard, SPSIPB may serve as an alternative to ESPB.^20^ The long-held perception of TEA as the ‘gold standard’ is increasingly being challenged. Literature reviews have concluded that TPVB provides postoperative analgesia comparable to TEA.^21^ A 2016 Cochrane study by Yeung et al. found moderate-quality evidence indicating similar analgesic efficacy between the two approaches. A protocol for managing postoperative pain after thoracotomy, including recommendations for regional analgesic methods, is available through PROSPECT.^22^ Furthermore, rhomboid intercostal block has been demonstrated to be a viable alternative for this purpose.

In 2023, Tulgar et al. introduced SPSIPB, a novel planar block that may serve as an alternative for postoperative pain management after thoracotomy due to its ease of application and effective analgesia.^8^ Cadaver studies have demonstrated that local anesthetics are widely used in the thoracic area. This suggests that local anesthesia may provide analgesia during VATS procedures. In a single prospective randomized study of 24 patients, Avcı et al. compared VATS patients who received SPSIPB + IV tramadol PCA with those who received IV tramadol PCA alone.^23^ They found that effective analgesia was achieved in patients who received SPSIPB. At the same time, the use of SPSIPB as a component of multimodal analgesia in postoperative pain management in thoracic surgery is increasing. In their case report, Celik et al.^24^ used SPSIPB as a component of multimodal analgesia in a patient with a clavicle fracture and achieved effective analgesia in the first eight hours of postoperative follow-up without the need for additional analgesic interventions. They also noted that the superficial and easily accessible nature of the SPSIPB application provided a significant advantage during its administration. In a separate study, Ciftci et al.^25^ initially planned to use ESPB as a regional anesthetic technique in pediatric patients undergoing posterior instrumentation between T2 and L1 for thoracic scoliosis. However, due to difficulty distinguishing transverse processes under US in the postoperative period, they administered SPSIPB instead. During the postoperative follow-up, the researchers reported a numerical rating score below 3 and noted that no additional analgesic drugs or opioids were required. These data suggest that SPSIPB may be a viable alternative for managing multimodal analgesia in patients undergoing thoracic surgery, given its ease of administration and efficacy. In our results, the overall analgesic effect and the incidence of side effects were similar, and our QoR-15 results were comparable. This suggests that the application of SPSIPB could serve as an alternative in multimodal analgesia management for VATS surgery.

Many studies have compared TPVB with other planar blocks with quite different results. Sertcakacılar et al. showed in their study that single-injection TPVB provided superior analgesia compared to ESPB in patients undergoing single-port VATS and demonstrated a greater opioid-sparing effect by reducing morphine consumption in the TPVB group.^26^ In contrast, Zengin et al. found lower VAS scores in the ESPB group compared to the TPVB group in a randomized controlled study comparing ESPB, TPVB, and the combination of ESPB + TPVB in VATS patients.^27^ Similarly, Çiftçi et al. found that both ESPB and TPVB provided more effective analgesia compared to the control group in VATS patients. They also noted that ESPB had a shorter duration of performance and a higher single-puncture success rate than TPVB.^28^ Other studies comparing SAPB with TPVB have indicated that SAPB can be safely and quickly used in VATS patients, providing analgesia as effective as TPVB and potentially serving as an alternative.^29^ While TPVB has been compared with SPSIPB in terms of postoperative analgesic efficacy in studies of breast surgery and minimally invasive cardiac surgery,^30^^,^^31^ to the best of our knowledge, there is no study in the literature comparing TPVB with SPSIPB in patients undergoing VATS. Our study results show that SPSIPB provides analgesic outcomes similar to TPVB in VATS patients. Although we did not evaluate duration, we can state that we observed a rapid application, which is in alignment with the literature. We attribute this ease of identification of anatomical structures to ultrasound, as is the case with other planar blocks.

One of the intriguing findings of our study, although not statistically significant, is that during the early postoperative period (first hour), opioid consumption was more restricted in the TPVB group, while it was lower in the SPSIPB group within 24 hours. It is well known that the analgesic effect of thoracic fascial blocks differs from that of TPVB. The dermatomal spread of regional anesthetic blocks plays an important role in their analgesic efficacy, especially in thoracic surgery. TPVB has been documented to provide analgesia by delivering local anesthetic into the paravertebral space, affecting both the dorsal and ventral rami of the spinal nerves.^17^ Typically, TPVB results in unilateral segmental spread from T2 to T6, though cadaver and infrared thermography tests have demonstrated dermatomal spread from T2 to T10, varying by volume and technique used.^32^^,^^33^ In contrast, the mechanism of action of the more recently introduced SPSIPB is more complicated. It provides analgesia by targeting the interfascial plane between the serratus posterior superior muscle and the intercostal nerves at the T2–T5 levels, with its effect extending along the upper intercostal and lower cervical nerves. Cadaver studies have shown spread between C7 and T7 levels.^8^ Although there are limited data in the literature, a dermatomal analysis study has also reported spread between C3 and T7.^8^ Unlike TPVB, which has a more predictable diffusion into the paravertebral space, SPSIPB primarily provides analgesia through interfascial diffusion, which may lead to variability in its mechanism of action. Future studies comparing the consistency of dermatomal spread between these two blocks could clarify their relative efficacy in thoracic analgesia. In TPVB, local anesthetic acts through the paravertebral space, affecting nerve roots and the epidural area.^17^ This allows for faster and more effective diffusion of analgesia compared to the thoracic paravertebral area’s potential space. However, the transition through the fascial pores that delimit the paravertebral space, as well as quick access to the epidural space and nerve roots, allows for rapid analgesic effects. Conversely, in planar blocks, the diffusion of local anesthetics applied to fascial planes is thought to occur via neurovascular bundles passing through the fascial layers.^34^ This could result in a more prolonged effect on dorsal and ventral nerves. Our findings suggest that limited opioid consumption at the 24 hour mark in the TPVB group supports this notion. Although the difference in opioid consumption did not achieve statistical significance, it may still hold clinical relevance by contributing to an overall opioid-sparing effect in the postoperative period. Notably, the VAS rest score was significantly lower in the TPVB group at 1 hour postoperatively, indicating a potential advantage in early-phase analgesia. However, this early difference did not persist at later time points and was not accompanied by reductions in overall opioid consumption, patient satisfaction, or quality of recovery scores. Therefore, while this short-term benefit may not alter routine practice, it could be clinically relevant in specific contexts such as early mobilization, physiotherapy, or postoperative imaging, where superior immediate pain control is advantageous. Together, these findings suggest that TPVB may offer superior early-phase analgesia, while SPSIPB may provide advantages in sustained analgesic efficacy and reduced opioid requirements over time. These observations illustrate the potential complementary roles of these techniques in multimodal analgesia strategies for thoracic surgery. Further studies with larger sample sizes and extended follow-up are warranted to corroborate these findings and elucidate their implications for long-term postoperative outcomes, including the incidence of chronic postsurgical pain.

In our study, in addition to assessing VAS scores, opioid consumption, and side effects, we also evaluated the QoR-15 questionnaire to ensure the reliability of the results. As is well known, the QoR-15 provides a multidimensional assessment of postoperative recovery, and the resulting scores are recommended as an endpoint in clinical studies focusing on postoperative pain.^35^ The QoR-15 is now increasingly being used as an effective tool in postoperative applications^36^ and is used to evaluate the effectiveness of Enhanced Recovery After Surgery (ERAS) protocols, which are being applied more frequently in thoracic anesthesia.^37^ The QoR-15 has indicated a significant correlation between postoperative pain and postoperative recovery.

Another important consideration in the clinical application of SPSIPB is the optimal volume of local anesthetic required to achieve effective analgesia. In the present study, a volume of 30 mL of 0.25% bupivacaine was used for SPSIPB to ensure adequate interfascial spread and dermatomal coverage. However, the optimal volume for this block technique remains unclear in the literature. Recent studies, including the report by Ciftci et al.,^9^ have demonstrated that lower volumes, such as 20 mL, may provide sufficient analgesic efficacy while potentially minimizing the risk of Local Anesthetic Systemic Toxicity (LAST). These findings suggest that further research is needed to determine whether reduced volumes can maintain analgesic efficacy while improving the safety profile of SPSIPB. Future randomized trials comparing different local anesthetic volumes may provide valuable evidence to optimize the balance between efficacy and safety for this novel regional technique.

Our study has several limitations. First, the absence of a control group prevented the evaluation of outcomes of both block techniques compared to a standard care group without regional anesthesia. Second, although the study was conducted at two centers, patient follow-up was standardized by assessing outcomes during the first 24 hours in the postoperative intensive care unit. However, a longer follow-up period could have provided additional insight into the prolonged effects of these blocks on acute postoperative pain. Furthermore, we did not evaluate chronic pain development. Another limitation is the lack of dermatome mapping, as sensory coverage was not evaluated after SPSIPB was performed; therefore, the extent of sensory blockade could not be confirmed. The potential influence of individual thoracic anatomy, BMI, and prior opioid exposure on block efficacy in our study population cannot be discounted; however, these variables were not analyzed in detail. Future studies should consider a stratified analysis to determine whether specific patient subgroups respond differently to SPSIPB compared to TPVB. Furthermore, although we used a standardized volume of local anesthetic, different volumes or concentrations may result in different analgesic outcomes. Finally, the duration of the sensory blockade and the evaluation of regression patterns were not assessed in this study.

## Conclusion

This randomized controlled trial demonstrated that SPSIPB provides postoperative analgesia comparable to that of TPVB in patients undergoing VATS. While TPVB was associated with lower resting pain scores during the early postoperative period, no significant differences were observed between the two groups in terms of overall pain scores, total opioid consumption, patient satisfaction, or QoR-15 outcomes within the first 24 hours postoperatively.

SPSIPB appears to be a promising alternative to TPVB for postoperative analgesia in minimally invasive thoracic surgery, offering a technically simpler, safer, and comparably effective option. Its superficial anatomical location and ease of administration may make it a particularly attractive choice in multimodal analgesia strategies. Further studies with larger sample sizes, extended follow-up, and more diverse surgical populations are warranted to validate these findings and to better define the role of SPSIPB in routine clinical practice.

## Declarations

Ethical approval: This study was approved by the Hitit University Faculty of Medicine Ethics Committee (Approval n° 2023-181).

ClinicalTrials.gov identifier: NCT06219369 (January 23, 2024).

Data access statement: The data that support the findings of this study are available from the corresponding author upon reasonable request.

## Data availability

The data that support the findings of this study are available from the corresponding author upon reasonable request.

## Authors’ contributions

Güvenç Doğan, Onur Küçük, Selçuk Kayır and Bahadır Çiftçi: Conception and design of the study. Güvenç Doğan, Musa Zengin and Ali Alagöz: Data acquisition. Gökçe Çiçek Dal, Selçuk Kayır, Ali Alagöz and Bahadır Çiftçi: Writing/manuscript preparation. Güvenç Doğan, Musa Zengin and Gökçe Çiçek Dal: Supervision.

## Funding

The study had no funding source.

## Conflicts of interest

The authors declare no conflicts of interest.
